# Global burden of maternal hypertensive disorders (1990–2045): trends, regional disparities, and causal links to occupational exposures

**DOI:** 10.1186/s12884-025-07766-y

**Published:** 2025-06-02

**Authors:** Junpeng Xiong, Shuwen Chen, Han Wang, Xiaonan Yang, Xinyi Chen, Binquan You, Ronghui Yu

**Affiliations:** 1 Department of Cardiology, National Cardiovascular Disease Regional Center for Anhui, The First Affiliated Hospital of Anhui Medical University, No. 120, Wanshui Road, Shushan District, Hefei, Anhui 230088 China; 2 Department of Neurology, Second Affiliated Hospital of Anhui Medical University, 678 Furong Road, Hefei, Anhui 230601 China; 3 Department of Otolaryngology Head and Neck Surgery, Fuyang Women and Children’s Hospital, No. 2019, Huaihe Road, Yingzhou District, Fuyang, Anhui 236001 China; 4 Department of Neurology, Fengtai County People’s Hospital, Fengtai County, No. 2 Huaibin Road, Beicheng District, Huainan, Anhui 232100 China; 5 Department of Cardiology, Suzhou Kowloon Hospital, Shanghai Jiaotong University School of Medicine, 118 Wansheng Street, Suzhou, Jiangsu 215000 China

**Keywords:** Maternal hypertensive disorders (MHD), Global burden disease (GBD), Cross-country inequality, Occupational exposures, Mendelian randomization (MR)

## Abstract

**Background:**

Maternal Hypertensive Disorders (MHD), encompassing gestational hypertension, chronic hypertension, preeclampsia, and eclampsia, which was a significant contributor to maternal morbidity and mortality, particularly in regions with lower socioeconomic status.

**Methods:**

Using data from the 2021 Global Burden of Disease (GBD) study, we analyzed the burden of MHD globally. We used the slope index and concentration index to measure cross-country inequality in MHD burden, and employed a Bayesian age-period-cohort (BAPC) model to project the burden from 2022 to 2045. Additionally, we conducted a two-sample Mendelian randomization (MR) analysis based on genome-wide association study (GWAS) to investigate potential causal relationships between occupational exposures and MHD.

**Results:**

Overall, the global incidence, prevalence, mortality, and DALYs for MHD have declined. However, incidence and prevalence rose in Central Asia, Eastern Europe, and Western Europe, while the Caribbean increasing in mortality and DALYs. Iron deficiency emerged as the leading risk factor. Significant SDI-related inequalities in MHD burden were observed, especially in lower SDI countries. Projections suggest ongoing reductions in MHD burden through 2045. MR results revealed a significant causal link between frequent exposure to chemical or other fumes in the workplace and MHD, while no clear causal relationships were identified for particulate matter or other assessed exposures.

**Conclusions:**

Although the global MHD burden is decreasing, marked regional disparities persist. Efforts focusing on addressing iron deficiency, improving nutritional support, and mitigating occupational exposures may further reduce the burden. Strengthening maternal healthcare services—especially in low-SDI will be crucial for achieving sustainable declines in MHD worldwide.

**Supplementary Information:**

The online version contains supplementary material available at 10.1186/s12884-025-07766-y.

## Graphical Abstract

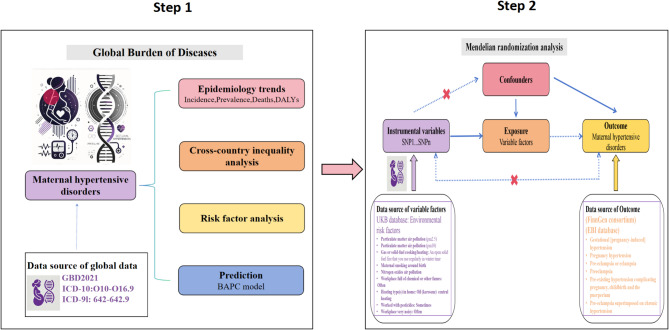

## Introduction

Maternal Hypertensive Disorders (MHD) are a major global public health concern. They include gestational hypertension, chronic hypertension, preeclampsia, eclampsia, and preeclampsia superimposed on chronic hypertension, collectively affecting approximately 5–15% of pregnant women worldwide [1, 2]. Research has shown that women who have experienced MHD—particularly those with eclampsia and severe preeclampsia—are linked to higher rates of all-cause mortality, perinatal mortality, cardiovascular disease mortality, digestive system disease mortality, as well as mortality from endocrine, nutritional, and metabolic diseases among their offspring from birth through adolescence [3–7]. Furthermore, the ongoing global burden of maternal hypertensive disorders (MHD), particularly in low- and middle-income countries, is associated with an increasing trend in long-term maternal morbidity and mortality [8].

Maternal hypertensive disorders (MHD) are caused by the interaction of multiple factors including genetic susceptibility, environmental and occupational exposures, pre-existing clinical conditions (chronic hypertension, diabetes), and sociodemographic factors (maternal age, parity, and access to health care) [3–11], underscoring the importance of further epidemiological research and policy support to improve maternal health and reduce global health inequalities. Air pollution is a major occupational health issue affecting populations in low-, middle-, and high-income countries. Although previous epidemiological and large-scale cohort studies indicate that occupational pollutants are risk factors for MHD, contributing to increased incidence and mortality [12–15], emerging evidence on environmental and occupational determinants of hypertension outcomes in pregnancy, including exposure to air pollutants, noise, and chemical fumes, remains underexplored in causal epidemiology. To address this gap, we conducted a two-sample Mendelian randomization study, using genome-wide genetic variations such as single-nucleotide polymorphisms (SNPs) as instrumental variables (IVs) to estimate the causal relationship between occupational exposures and MHD.

Using the 2021 Global Burden of Disease (GBD) database, we evaluated the trends and burden of MHD from 1990 to 2021, examined health inequalities among countries in different regions, analyzed its attributable risk factors, and projected the disease burden from 2022 to 2045. We further explored the potential causal relationship between air pollutants and gestational hypertension through Mendelian randomization, thereby enhancing our understanding of air pollution as a risk factor. Our study provides the most up-to-date epidemiological data on MHD worldwide, offering profound insights into the promotion of maternal and infant health and the achievement of global health goals. This analysis not only informs healthcare professionals and policymakers but also assists governments worldwide in formulating maternal health policies.

## Methods

### Study data

The GBD analysis process of this study is shown in Step 1 in the Central Illustration. The burden data originated from the GBD 2021. The GBD 2021 database, which serves as a public resource, is supplied by the Institute for Health Metrics and Evaluation of the University of Washington [16]. The GBD 2021 study conducts a thorough assessment of health consequences related to 371 diseases and injuries [17]. We produced estimates for 204 countries and territories that were grouped into 21 regions and seven super-regions (Table 1). The seven super-regions are central Europe, eastern Europe, and central Asia; high income; Latin America and the Caribbean; north Africa and the Middle East; south Asia; southeast Asia, east Asia, and Oceania; and sub-Saharan Africa. In GBD 2021 we continue to analyse at subnational levels countries that were added in previous cycles including Brazil, China, Ethiopia, India, Indonesia, Iran, Italy, Japan, Kenya, Mexico, New Zealand, Nigeria, Norway, Pakistan, Russia, the Philippines, Poland, South Africa, Sweden, the UK, and the USA. The GBD 2021 study uses the Bayesian meta-regression tool DisMod-MR 2.1 as its core model to estimate the burden of disease across countries, years, age groups, and sexes. The model integrates data from a wide range of sources (including surveys, registries, hospital records, and demographic data) while adjusting for known biases (e.g., misclassification or underreporting). It assumes internal consistency among key epidemiological indicators (incidence, prevalence, remission, and mortality) and applies differential equations to ensure consistency. When original data are sparse or missing, covariate-based predictions are used to take advantage of data from similar countries, regions, or years. Its study design and methodology have been extensively detailed in existing GBD literature (https://ghdx.healthdata.org/gbd-results‐tool).

**Table 1:** The case number and ASR of Incidence of MHD in 1990 and 2021 for female by SDI quintiles and by GBD regions, with EAPC from 1990 to 2021

Incidence	1990		2021		
	Number (95% UIs)	ASIR(95% UIs)	Number (95% UIs)	ASIR(95% UIs)	EAPC (95% CI) 1990–2021
Global	15,662,895(12,935,145 − 19,254,513)	919.34(759.23-1,130.15)	18,050,085(15,356,124 − 21,519,204)	723.53(615.54-862.59)	-0.51 (-0.56 to -0.45)
High SDI	1,233,522(966,651-1,612,143)	440.55(345.24-575.78)	1,235,244(1,023,404-1,513,215)	399.58(331.05–489.50)	-0.49 (-0.67 to -0.31)
High-middle SDI	1,488,518(1149,315-1,967,034)	427.48(330.07-564.91)	1,249,379(1,008,834-1,562,214)	319.54(258.02-399.55)	0.05 (-0.31 to 0.41)
Middle SDI	4,056,969(3,303,930-5,098,008)	716.47(583.48-900.31)	3,701,256(3,110,329-4,468,478)	468.48(393.68-565.59)	-0.72 (-0.88 to -0.57)
Low-middle SDI	4,734,331(3,973,547-5,762,420)	1,320.24(1,108.08-1,606.94)	5,103,004(4,291,790-6,055,102)	794.26(668-942.45)	-1.83 (-1.99 to -1.68)
Low SDI	4,139,304(3,487,342-4,867,143)	2,776.52(2,339.20-3,264.73)	6,749,882(5,727,623-7,955,523)	1,874.36(1,590.49-2,209.15)	-1.28 (-1.4 to -1.16)
Andean Latin America	60,880(54,015–69,612)	492.72(437.16-563.39)	102,409(95,875 − 110,222)	468.01(438.15-503.72)	-0.16 (-0.36 to 0.04)
Australasia	35,035(29,359 − 42,314)	532.62(446.32-643.28)	33,846(26,068 − 42,709)	369.09(284.26-465.73)	-1.03 (-1.23 to -0.84)
Caribbean	93,720(74,276 − 120,931)	796.5(631.26-1027.76)	83,402(67,141 − 104,886)	545.37(439.04-685.86)	-0.95 (-1.02 to -0.87)
Central Asia	70,849(56,869 − 89,435)	323.69(259.82-408.61)	91,670(73,030–116,744)	298.04(237.44-379.56)	0.4 (0.11 to 0.7)
Central Europe	112,704(83,118–154,106)	285.66(210.67-390.59)	80,855(66,374 − 100,780)	247.17(202.90-308.08)	-0.29 (-0.64 to 0.06)
Central Latin America	684,950(574,021–824,555)	1,258.84(1,054.97-1,515.42)	621,918(545,662–717,391)	722.16(633.61-833.02)	-0.99 (-1.33 to -0.65)
Central Sub-Saharan Africa	563,799(466,899 − 670,890)	3,413.33(2,826.687-4,061.68)	878,783(721,967-1,077,617)	2,022.87(1,661.90-2,480.57)	-1.51 (-1.63 to -1.39)
East Asia	1,278,231(942,274-1,787,350)	313.09(230.80-437.80)	710,146(548,939 − 920,215)	163.57(126.45-211.98)	-0.57 (-1.7 to 0.58)
Eastern Europe	429,926(320,324–586,671)	597.86(445.45-815.83)	372,397(286,377–479,586)	608.35(467.82-783.45)	1.12 (0.65 to 1.59)
Eastern Sub-Saharan Africa	1,976,139(1,638,189-2,341,423)	3,414.77(2,830.79-4,045.98)	3,087,073(2,599,544-3,620,626)	2,204.79(1,856.59-2,585.85)	-1.39 (-1.52 to -1.26)
High-income Asia Pacific	179,021(141,199–232,968)	312.17(246.21-406.23)	115,278(98,177 − 135,473)	234.77(199.94-275.89)	-1.52 (-1.99 to -1.05)
High-income North America	581,683(445,278–766,637)	643.34(492.47-847.89)	626,403(539,595–736,474)	584.1(503.15-686.72)	-0.48 (-0.71 to -0.25)
North Africa and Middle East	1,091,557(866,925-1,390,333)	1,055.518(838.301-1,344.43)	1,205,613(946,504-1,540,737)	597.84(469.35-764.02)	-1.49 (-1.66 to -1.33)
Oceania	17,955(14,074 − 23,015)	887.73(695.81-1,137.90)	33,574(26,578 − 42,939)	758.02(600.05-969.45)	-0.72 (-0.76 to -0.67)
South Asia	4,018,273(3,329,687-4,920,134)	1,207.95(1,000.95-1,479.06)	3,478,494(2,875,246-4,288,315)	559.77(462.69-690.09)	-2.8 (-3.17 to -2.44)
Southeast Asia	1,195,303(945,536-1,507,583)	770.38(609.40-971.64)	1,143,820(917,858-1,420,431)	493.36(395.89-612.66)	-1.33 (-1.39 to -1.26)
Southern Latin America	107,883(82,156 − 140,036)	678.62(516.79-880.87)	134,877(113,450 − 162,874)	616.64(518.68-744.64)	-0.1 (-0.2 to 0.01)
Southern Sub-Saharan Africa	342,754(283,702 − 409,228)	1,979.57(1,638.52-2,363.49)	366,803(307,516 − 431,853)	1,339.08(1,122.64-1,576.55)	-1.09 (-1.14 to -1.03)
Tropical Latin America	413,752(331,184–535,494)	804.11(643.65-1,040.71)	375,061(321,710 − 447,913)	497.09(426.38-593.64)	-1.22 (-1.48 to -0.96)
Western Europe	372,227(285,328–488,578)	312.83(239.80-410.62)	387,665(309,193–488,869)	321.12(256.12-404.95)	0.41 (0.27 to 0.54)
Western Sub-Saharan Africa	2,036,247(1,723,735-2,377,706)	3,508.93(2,970.40-4,097.34)	4,119,997(3,534,414-4,761,707)	2,593.41(2,224.81-2,997.35)	-0.84 (-1.03 to -0.66)

Abbreviations: ASIR, age-standardized incidence rate; MHD, Maternal Hypertensive Disorders; SDI, sociodemographic index; GBD, Global Burden of Diseases, Injuries, and Risk Factors Study; EAPC, estimated annual percentage change; UIs, uncertainty intervals; CI, confidence interval

### Indicator Definitions

According to the International Classification of Diseases, Ninth Revision (ICD-9: 642–642.9) and Tenth Revision (ICD-10: O10–O16.9), Maternal Hypertensive Disorders (MHD) are defined as follows: (1)Pre-existing hypertension complicating pregnancy, childbirth and the puerperium.(2)Pre-existing hypertension with pre-eclampsia.(3)Gestational [pregnancy-induced] edema and proteinuria without hypertension.(4)Gestational [pregnancy-induced] hypertension without significant proteinuria.(5)Pre-eclampsia.(6)Eclampsia. (7)Unspecified maternal hypertension. Mapping was performed using the GBD etiology hierarchy to ensure consistency across data sources and years. GBD defines each disease using a standardized algorithm applied to administrative data, surveys, and vital registries.

### Data acquisition and preliminary analysis

The 95% uncertainty intervals (UI) for incidence, prevalence, mortality, and disability-adjusted life years (DALYs) are derived directly from the GBD 2021 estimates based on 1,000 posterior draws generated by a Bayesian meta-regression model (DisMod-MR 2.1). New cases and incident cases were distinguished from prevalent cases based on the temporal patterns of diagnostic coding and record structure. However, we acknowledge that some degree of misclassification (possibly including recurrent cases) is possible due to the limitations of routinely collected data. These draws reflect uncertainty from multiple sources, including variability in the input data, sampling error, and model assumptions. For each health indicator, the 2.5 and 97.5 percentiles of the 1,000 draws were used to construct the 95% UI, ensuring that the probabilities reflect the range of estimates. The estimated annual percentage change (EAPC) was computed by employing a least squares linear regression model, and the results of the regression were organized and analyzed using the R package. For Estimated Annual Percentage Change (EAPC) calculations, we fit a linear regression model to the natural logarithm of the age-standardized rates over time. The 95% CIs of the EAPCs were derived from the standard error of the regression coefficient, assuming a normal distribution [18]. It is computed using the formula: ln(y) = α + βx + ε, where (y) stands for the age-standardized incidence, (α) is the intercept, (x) represents the year, (β) is the slope, and (ε) is the normally distributed error term. The EAPC formula is (exp^β − 1)×100%. An upward trend in age-standardized incidence is shown when the lower bound of the EAPC 95% confidence interval (CI) is greater than 0; a downward trend is indicated when the upper bound of the EAPC 95% CI is less than 0; otherwise, the incidence is considered relatively stable. For Mendelian randomization (MR) estimates, 95% CIs were calculated using the delta method or standard error propagation methods, depending on the MR method applied (e.g., IVW, MR Egger) [19]. In order to analyze the global distribution and regional differences in MHD, the generated global maps were conducted regional comparative analyses. Data were aggregated according to the geographic regions defined by the GBD study to visualize the geographic distribution of the disease burden. Based on population to explore the distribution of MHD across age groups. Additionally, the relationship between the SDI and MHD burden were investigated. The SDI classification (low, low-middle, middle, high-middle, and high) was used to compare disease burden across different levels of socioeconomic development [20].

### Cross-country inequality analysis

Health inequality monitoring can lay a foundation for evidence-based health planning and further refine relevant policies, programs, and practices in order to reduce differences in health distribution. In the present study, two standard metrics for absolute and relative gradient inequality, specifically the slope index of inequality and the concentration index, were employed to measure the inequality in the distribution of the Maternal Hypertensive Disorders (MHD) burden across countries. The slope index of inequality was determined by regressing national DALYs rates of all age populations on a sociodemographic development-related relative position scale. The concentration index was calculated by numerically integrating the area under the Lorenz concentration curve, which was fit using the cumulative fraction of DALYs and the cumulative relative distribution of the population ranked by SDI [18].

### Prediction analysis

Using a Bayesian age-period-cohort (BAPC) analysis based on the 1990–2021 data from the Global Burden of Disease (GBD) database for global AF/AFL incidence, prevalence, mortality, and DALYs, we projected the future burden of MHD for the period 2022–2045. Population estimates were obtained from the 2019 revision of the United Nations World Population Prospects, disaggregated by year (up to 2100), age, and sex (https://population.un.org/wpp/downloads). The BAPC model builds on the age-period-cohort (APC) framework—a model commonly used to analyze trends in the incidence and mortality of chronic diseases—and employs the integrated nested Laplace approximation (INLA) algorithm for parameter estimation [21]. The classic APC model describes changes in incidence or mortality as functions of age, period, and cohort, thus enabling predictions based on these trends [22].

### MR analysis

In this study, the Mendelian randomization (MR) flowchart is presented in Step 2 of the Central Illustration. This Mendelian randomization analysis was conducted and reported in accordance with the STROBE-MR (Strengthening the Reporting of Observational Studies in Epidemiology Using Mendelian Randomization) guidelines [23], and the STORBE-MR checklist is provided in the Supplementary Table 22. We applied an MR approach combined with genome-wide association study (GWAS) data to investigate the potential causal relationship between occupational exposures and Maternal Hypertensive Disorders (MHD). Specifically, particulate matter was regarded as the exposure variable (Supplementary Table 1), while MHD was considered the outcome variable (Supplementary Table 2). Exposure data on occupational pollutants and MHD case data were obtained from the UK Biobank and FinnGen databases, respectively, covering participants in both cohorts. We identified independent SNPs associated with particulate matter based on two criteria. First, we selected SNPs at a genome-wide significance threshold of *p* < 5 × 10^(-6). Second, we used pairwise linkage disequilibrium (LD) analyses to assess SNP independence, selecting SNPs that were at least 10,000 kb apart and excluding those with LD (R^2 < 0.01). The instrumental variables used in this study are listed in Supplementary Table 3. To bolster the reliability of our analysis, we applied five different methods: MR-Egger, weighted median, inverse variance weighting (IVW), simple mode, and weighted mode. Among these, IVW was our primary approach because—when no horizontal pleiotropy exists among the instrumental variables—it provides the most robust evidence [24]. However, if horizontal pleiotropy is present, other causal pathways beyond the exposure of interest may confound the results. Thus, we employed the other four complementary methods to ensure that our findings remained robust to potential confounding factors. We then compared the conclusions from all five methods to evaluate consistency and reliability. To further validate the robustness of our results, we performed sensitivity analyses including the MR-Egger intercept test and MR pleiotropy residual analysis [25]. Additionally, we conducted leave-one-out analyses and created scatter plots to visually identify and examine potential outliers.

### Statistical analysis

The projected absolute figures for each metric spanning from 2022 to 2045 were computed by multiplying the yearly age-sex-country-specific projection ratios with the population projections of the corresponding subgroups within the same year. These estimates were then combined to showcase the outcomes at the global scale, as well as at the World Bank income group levels and on a country-by-country basis. To calculate the age-adjusted rates for incidence, prevalence, mortality, and DALYs, the direct standardization technique was employed, with the World Health Organization standard population serving as the benchmark. Eventually, the average annual percentage alteration and associated 95% confidence interval for each measure during the period from 2022 to 2045 were determined to gauge the shift in the global age-adjusted rates. All of the analytical procedures were carried out using the R statistical software (version 4.3.2). MR analysis was performed using the R packages “TwoSampleMR” and “data.table”.

## Result

### Analysis of the trend of global disease burden of MHD

**Fig. 1:**
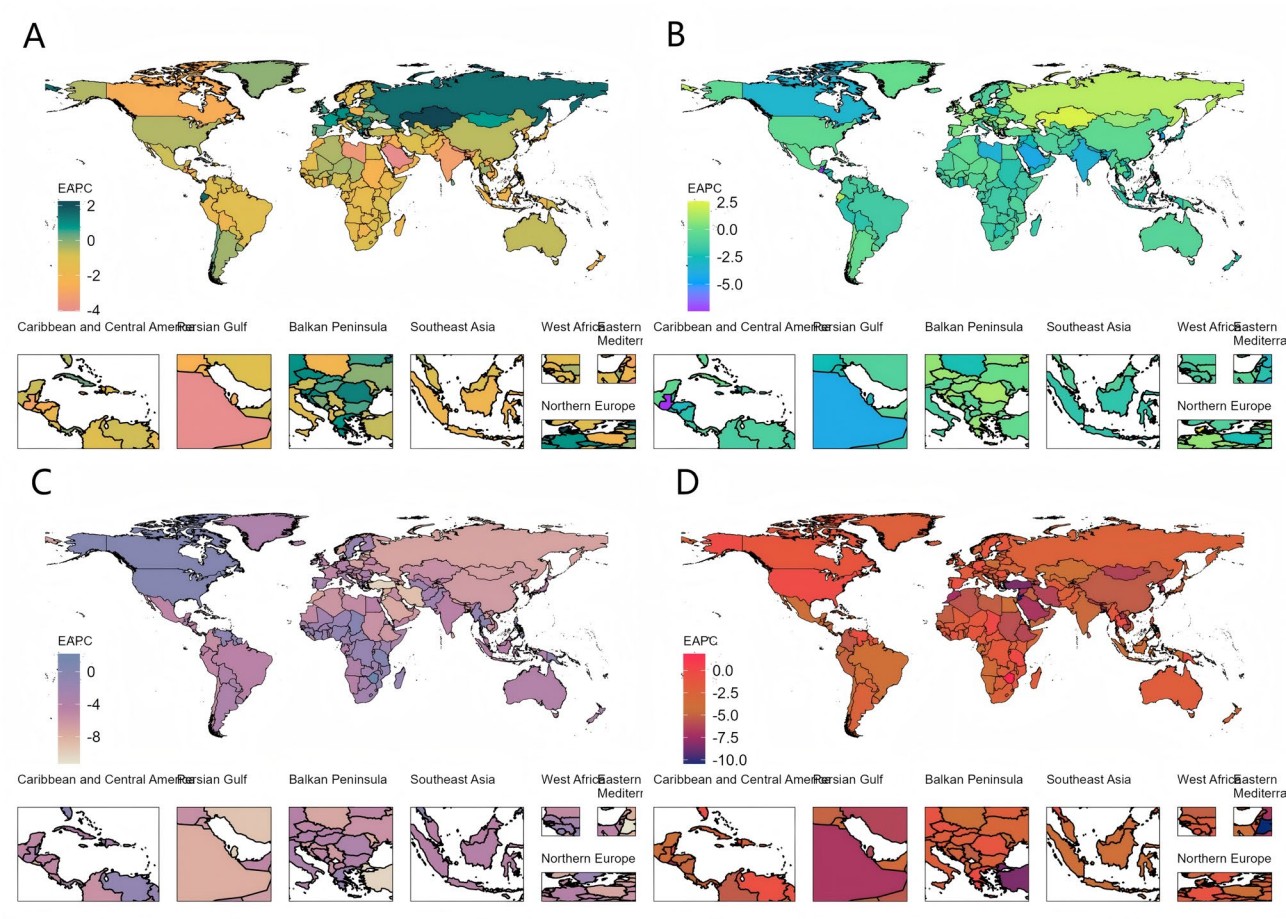
The trend in ASR of EAPC from 1990 to 2021. **A** The trend in ASR of incidence (EAPC) from 1990 to 2021; **B** The trend in ASR of prevalence (EAPC) from 1990 to 2021; **C** The trend in ASR of deaths (EAPC) from 1990 to 2021; **D** The trend in ASR of DALYs (EAPC) from 1990 to 2021, of MHD worldwide. Abbreviations: ASR, age-standardized rate; EAPC, estimated annual percentage change; DALYs, disability-adjusted life-years; MHD, Maternal Hypertensive Disorders

Analyses of the Global Burden of Disease (GBD) database indicate a clear trend in the worldwide epidemiology of Maternal Hypertensive Disorders (MHD). While the total number of incident cases increased from 15,662,895 (95% UI: 12,935,146–19,254,514) in 1990 to 18,050,085 (95% UI: 15,356,124–21,519,204) in 2021, the incidence rate per 100,000 population decreased from 919.34% (95% UI: 759.23%–1,130.15%) in 1990 to 723.53% (95% UI: 615.54–862.59%) in 2021, marking an overall decline of 21.3%. The estimated annual percentage change (EAPC) was − 0.51% (95% CI: -0.56 to -0.45) (Table 1; Fig. 1A, Supplementary Fig. 1A-C). However, in Central Asia, Eastern Europe, and Western Europe, incidence rates moved in the opposite direction of the global trend, with EAPCs of 0.40% (95% CI: 0.11 to 0.70), 1.12% (95% CI: 0.65 to 1.59), and 0.41% (95% CI: 0.27 to 0.54), respectively (Table 1; Fig. 1A).

Consistent with incidence, the overall prevalence of MHD declined over the past 30 years. Although the total number of prevalent cases rose from 3,076,691 (95% UI: 1,997,627–4,481,074) in 1990 to 3,520,625 (95% UI: 2,279,278–5,084,397) in 2021, the prevalence rate decreased from 180.59% (95% UI: 117.25–263.02) per 100,000 in 1990 to 141.12% (95% UI: 91.36–203.81) in 2021, for an overall reduction of 21.90%. The EAPC was − 0.54% (95% CI: -0.60 to -0.47) (Table 2; Fig. 1B, Supplementary Fig. 1D-F). Nonetheless, in Central Asia, Eastern Europe, and Western Europe, prevalence showed an upward trend contrary to the global pattern, with EAPC of 0.49% (95% CI: 0.17 to 0.81), 1.13% (95% CI: 0.66 to 1.60), and 0.38% (95% CI: 0.22 to 0.54), respectively (Table 2; Fig. 1B).

**Table 2:** The case number and ASR of prevalence of MHD in 1990 and 2021 for female by SDI quintiles and by GBD regions, with EAPC from 1990 to 2021

Prevalence	1990		2021		
	Number (95% UIs)	ASPR(95% UIs)	Number (95% UIs)	ASPR(95% UIs)	EAPC (95% CI) 1990–2021
Global	3,076,691 (1,997,627-4,481,074)	180.59 (117.25-263.02)	3,520,625 (2,279,278-5,084,397)	141.12 (91.36-203.81)	-0.54 (-0.6 to -0.47)
High SDI	261,686 (167,014–391,787)	93.46 (59.65-139.93)	259,630 (168,010–377,648)	83.99 (54.35–22.16)	-0.51 (-0.7 to -0.32)
High-middle SDI	328,87 (206,50–499,19	94.34(59.41–3.54)	281,971 (176,919 − 418,676)	72.12 (45.25-107.08)	0.15 (-0.21 to 0.51)
Middle SDI	817,326 (526,616-1,201,473)	144.34 (93.00-212.18)	734,683 (467,492 1,071,245)	92.99 (59.17-135.59)	-0.79 (-0.94 to -0.63)
Low-middle SDI	861,716 (555,045 − 1,267,717)	240.30 (154.78-353.52)	939,109 (610,340-1,360,617	146.17 (94.00-211.77)	-1.89 (-2.1 to -1.68)
Low SDI	805,351 (529,167-1,171,500)	540.21 (354.95-785.81)	1,302,927 (860,960-1,902,981)	361.81 (239.08-528.43)	-1.3 (-1.44 to -1.15)
Andean Latin America	10,374 (6,930 − 14,915)	83.96 (56.09-120.71)	18,220 (12,690 − 25,001)	83.26 (57.99-114.26)	-0.16 (-0.49 to 0.17)
Australasia	8,037 (5,349 − 11,686)	122.19 (81.32-177.66)	7,409 (4,517 − 10,925)	80.79 (49.26-119.13)	-1.19 (-1.42 to -0.97)
Caribbean	18,586 (11,972 − 29,352)	157.96 (101.74-249.46)	15,638 (9,868 − 23,418)	102.26 (64.53-153.13)	-1.15 (-1.23 to -1.06)
Central Asia	14,389 (8,962 − 22,087)	65.74 (40.95-100.91)	19,104 (11,959 − 28,730)	62.11 (38.88–93.41)	0.49 (0.17 to 0.81)
Central Europe	25,131 (15,405 − 39,897)	63.7 (39.04-101.12)	17,869 (11,497 − 26,001)	54.62 (35.15–79.48)	-0.34 (-0.7 to 0.02)
Central Latin America	115,079 (74,433 − 168,867)	211.5 (136.8-310.35)	102,308 (68,856 − 145,556)	118.8 (79.95-169.02)	-0.82 (-1.23 to -0.4)
Central Sub-Saharan Africa	114,184 (75,279 − 167,133)	691.29 (455.75-1,011.85)	176,527 (114,494 − 257,551)	406.35 (263.55-592.86)	-1.55 (-1.72 to -1.38)
East Asia	276,325 (166,315–440,395)	67.68 (40.74-107.87)	156,013 (94,867 − 238,503)	35.94 (21.85–54.94)	-0.46 (-1.74 to 0.84)
Eastern Europe	102,064 (62,996 − 156,566)	141.93 (87.60-217.72)	88,184 (54,981 − 134,308)	144.06 (89.82-219.41)	1.13 (0.66 to 1.6)
Eastern Sub-Saharan Africa	388,816 (255,009-569813)	671.88 (440.66-984.64)	602,266 (396,782–876,263)	430.14 (283.38-625.83)	-1.41 (-1.57 to -1.25)
High-income Asia Pacific	31,578 (19,282 − 48,836)	55.06 (33.62–85.15)	18,741 (12,085 − 27,290)	38.17 (24.61–55.58)	-1.93 (-2.51 to -1.34)
High-income North America	124,115 (77,999 − 192,941)	137.27 (86.27-213.39)	131,064 (85,938 − 185,150)	122.21 (80.13-172.65)	-0.49 (-0.73 to -0.26)
North Africa and Middle East	239,480 (151,545 − 352,545)	231.57 (146.54-340.91)	270,126 (166,026-405191)	133.95 (82.33-200.93)	-1.4 (-1.57 to -1.23)
Oceania	3732 (2,366-5,547)	184.502 (116.969-274.256)	6,866 (4,293 − 10,429)	155.02 (96.92-235.46)	-0.78 (-0.82 to -0.74)
South Asia	690,077 (431,663-1,057,306)	207.45 (129.77-317.84)	597,394 (374,209–901,826)	96.13 (60.22-145.12)	-3.06 (-3.58 to -2.55)
Southeast Asia	250,255 (156,168–370,483)	161.29 (100.65-238.78)	245,050 (153,080–364,422)	105.7 (66.03-157.18)	-1.26 (-1.34 to -1.19)
Southern Latin America	24,774 (15,451 − 37,691)	155.84 (97.19-237.09)	31,021 (20,449 − 45,051)	141.83 (93.49-205.97)	-0.09 (-0.2 to 0.02)
Southern Sub-Saharan Africa	72,228 (47,321 − 104,626)	417.15 (273.3-604.27)	74,400 (48,546 − 106,736)	271.61 (177.22-389.66)	-1.19 (-1.25 to -1.13)
Tropical Latin America	75,485 (47,196 − 119,826)	146.70 (91.72-232.88)	65,638 (43,014–94,496)	86.99 (57.01-125.24)	-1.5 (-1.89 to -1.12)
Western Europe	82,654 (51,188 − 127,530)	69.46 (43.02-107.18)	85,626 (53,336 − 127,824)	70.93 (44.18-105.88)	0.38 (0.22 to 0.54)
Western Sub-Saharan Africa	409,329 (272,978 − 588,062)	705.37 (470.41-1,013.37)	791,163 (528,610-1,132,040)	498.01 (332.74-712.58)	-0.89 (-1.12 to -0.65)

Abbreviations: ASPR, age-standardized prevalence rate; MHD, Maternal Hypertensive Disorders; SDI, sociodemographic index; GBD, Global Burden of Diseases, Injuries, and Risk Factors Study; EAPC, estimated annual percentage change; UIs, uncertainty intervals; CI, confidence interval

Following the same pattern as incidence and prevalence, overall mortality and DALYs for MHD declined in the past 30 years. The number of deaths decreased by 29.20%, from 53,894 (95% UI: 47,676–59,819) in 1990 to 38,147 (95% UI: 31,879–46,096) in 2021. Meanwhile, DALYs dropped by 29.00%, from 3,479,885 (95% UI: 3,085,882–3,873,201) in 1990 to 2,469,636 (95% UI: 2,083,398–2,958,212) in 2021. The mortality rate per 100,000 population declined from 3.16% (95% UI: 2.80–3.51) in 1990 to 1.53% (95% UI: 1.28–1.85) in 2021, representing an overall reduction of 56.70%. The EAPC for mortality was − 2.16% (95% CI: -2.21 to -2.10) (Table 3; Fig. 1C, Supplementary Fig. 1G-I). The DALY rate per 100,000 population decreased by 51.50%, from 204.25% (95% UI: 181.13–227.34) in 1990 to 98.99% (95% UI: 83.51–118.58) in 2021, yielding an EAPC of -2.10% (95% CI: -2.15 to -2.04) (Table 4; Fig. 1D, Supplementary Fig. 1J-L). However, in the Caribbean region, mortality and DALYs showed an upward trend contrary to the global pattern, with EAPC of 0.80% (95% CI: 0.58 to 1.02) and 0.75% (95% CI: 0.53 to 0.96), respectively (Tables 3 and 4). For detailed country-specific trends in the MHD burden, please refer to Supplementary Tables 4–7.

**Table 3:** The case number and ASR of deaths of MHD in 1990 and 2021 for female by SDI quintiles and by GBD regions, with EAPC from 1990 to 2021

Deaths	1990		2021		
	Number (95% UIs)	ASDR(95% UIs)	Number (95% UIs)	ASDR(95% UIs)	EAPC (95% CI) 1990–2021
Global	53,895 (47,676 − 59,819)	3.16 (2.8–3.51)	38,147 (31,879 − 46,096)	1.53 (1.28–1.85)	-2.16 (-2.21 to -2.1)
High SDI	338 (285–397)	0.12 (0.10–0.14)	135 (114–163)	0.04 (0.04–0.05)	-2.71 (-2.87 to -2.55)
High-middle SDI	2,022 (1,625-2,412)	0.58 (0.47–0.69)	361 (300–448)	0.09 (0.08–0.12)	-5.33 (-5.45 to -5.21)
Middle SDI	10,549 (9,346 − 11,815)	1.86 (1.65–2.09)	4,961 (4,235-5,981)	0.63 (0. 54-0.76)	-3.22 (-3.29 to -3.15)
Low-middle SDI	23,080 (19,957 − 25,795)	6.44 (5.57–7.19)	14,069 (11,322 − 17,616)	2.19 (1.76–2.74)	-3.46 (-3.55 to -3.37)
Low SDI	17,872 (15,191 − 20,320)	11.99 (10.19–13.63)	18,588 (15,234 − 22,700)	5.16 (4.23–6.30)	-2.6 (-2.7 to -2.5)
Andean Latin America	643 (548–749)	5.20 (4.44–6.06)	337 (248–444)	1.54 (1.14–2.03)	-4.29 (-4.53 to -4.04)
Australasia	4 (3–5)	0.07 (0.05–0.08)	1 (1–2)	0.02 (0.01–0.02)	-3.88 (-4.51 to -3.25)
Caribbean	383 (307–483)	3.25 (2.61–4.10)	511 (326–750)	3.34 (2.13–4.91)	0.8 (0.58 to 1.02)
Central Asia	252 (224–283)	1.15 (1.02–1.3)	80 (66–97)	0.26 (0.22–0.31)	-4.53 (-4.84 to -4.22)
Central Europe	52 (45–60)	0.13 (0.11–0.15)	7 (6–8)	0.02 (0.02–0.03)	-4.88 (-5.3 to -4.44)
Central Latin America	1,128 (1,030 − 1,233)	2.07 (1.89–2.27)	495 (402–605)	0.57 (0.47–0.7)	-3.92 (-4.15 to -3.69)
Central Sub-Saharan Africa	2,464 (1,735-3,273)	14.92 (10.50-19.82)	2,679 (1,860-3,719)	6.17 (4.28–8.56)	-2.08 (-2.4 to -1.77)
East Asia	1,822 (1,245-2,462)	0.45 (0.31–0.60)	194 (140–268)	0.05 (0.03–0.06)	-6.52 (-6.78 to -6.26)
Eastern Europe	185 (159–211)	0.26 (0.22–0.29)	17 (14–21)	0.03 (0.02–0.03)	-6.67 (-7.02 to -6.31)
Eastern Sub-Saharan Africa	7,510 (6,429-8,642)	12.98 (11.11–14.93)	6,404 (5,098 − 7,877)	4.57 (3.64–5.63)	-3.24 (-3.38 to -3.09)
High-income Asia Pacific	53 (43–62)	0.092 (0.075–0.108)	7 (6–8)	0.01 (0.01–0.02)	-5.65 (-5.99 to -5.31)
High-income North America	88 (72–108)	0.1 (0.08–0.12)	82 (66–102)	0.08 (0.06–0.1)	0.12 (-0.16 to 0.4)
North Africa and Middle East	4,350 (3,443-5,186)	4.21 (3.33–5.01)	1,654 (1,188-2,209)	0.82 (0.59–1.1)	-5.12 (-5.3 to -4.93)
Oceania	43.604 (24.091–64.759)	2.16 (1.19–3.20)	79 (54–110)	1.78 (1.21–2.49)	-0.57 (-0.77 to -0.38)
South Asia	20,401 (17,206 − 23,202)	6.13 (5.17–6.97)	11,967 (8,863 − 15,766)	1.93 (1.43–2.54)	-3.92 (-4.11 to -3.74)
Southeast Asia	6,405 (5,359-7,510)	4.13 (3.45–4.84)	3,097 (2,487-3,898)	1.34 (1.07–1.68)	-3.44 (-3.52 to -3.36)
Southern Latin America	114 (97–135)	0.72 (0.61–0.85)	37 (29–46)	0.17 (0.13–0.21)	-3.65 (-3.96 to -3.34)
Southern Sub-Saharan Africa	623 (473–820)	4 (3–4)	475 (363–614)	1.73 (1.33–2.24)	-0.98 (-2.08 to 0.13)
Tropical Latin America	1,225 (1,093 − 1,373)	2.38 (2.12–2.67)	345 (299–393)	0.46 (0.4–0.52)	-4.44 (-4.88 to -4)
Western Europe	87 (77.- 98)	0.07 (0.07–0.08)	20 (17–22)	0.02 (0.01–0.02)	-3.99 (-4.28 to -3.69)
Western Sub-Saharan Africa	6,064 (4,654-7,378)	10.45 (8.02–12.71)	9,660 (7,354 − 12,669)	6.08 (4.63–7.97)	-1.51 (-1.6 to -1.43)

Abbreviations: ASDR, age-standardized deaths rate; MHD, Maternal Hypertensive Disorders; SDI, sociodemographic index; GBD, Global Burden of Diseases, Injuries, and Risk Factors Study; EAPC, estimated annual percentage change; UIs, uncertainty intervals; CI, confidence interval

**Table 4:** The case number and ASR of dalys of MHD in 1990 and 2021 for female by SDI quintiles and by GBD regions, with EAPC from 1990 to 2021

DALYs	1990		2021		
	Number (95% UIs)	ASDAR(95% UIs)	Number (95% UIs)	ASDAR(95% UIs)	EAPC (95% CI) 1990–2021
Global	3,479,885 (3,085,882-3,873,201)	204.25 (181.13-227.34)	2,469,636 (2,083,398-2,958,212)	98.99 (83.51-118.58)	-2.10 (-2.15 to -2.04)
High SDI	33,250 (26,096 − 42,851)	11.88 (9.32–15.30)	20,491 (14,260 − 29,256)	6.63 (4.61–9.46)	-1.70 (-1.81 to -1.6)
High-middle SDI	139,303 (112,428 − 168,990)	40.01 (32.29–48.53)	3488,4 (27,185 − 44,882)	8.92 (6.95–11.48)	-4.19 (-4.27 to -4.10)
Middle SDI	690,767 (607,609–780,248)	121.99 (107.30-137.79)	329,586 (282,911 − 391,530)	41.72 (35.81–49.56)	-3.05 (-3.12 to -2.99)
Low-middle SDI	1,480,228 (1,288,978-1,643,500)	412.78 (359.45-458.32)	888,004 (717,495-1,101,565)	138.21 (111.68-171.45)	-3.49 (-3.58 to -3.39)
Low SDI	1,134,178 (960,803-1,289,414)	760.77 (644.48–864.90)	1,194,634 (983,739-1,454,751)	331.74 (273.17-403.97)	-2.57 (-2.67 to -2.47)
Andean Latin America	39,982 (34,224 − 46,689)	323.59 (276.98-377.87)	20,884 (15,654 − 27,162)	95.44 (71.54-124.13)	-4.19 (-4.41 to -3.96)
Australasia	655(471–921)	9.96 (7.17–14.01)	444.13 (270.34-699.83)	4.84 (2.95–7.63)	-2.08 (-2.32 to -1.85)
Caribbean	24,004 (19,494 − 29,614)	204.00 (165.67-251.68)	30,763 (19,909 − 44,682)	201.16 (130.19-292.18)	0.75 (0.53 to 0.96)
Central Asia	16,051 (14,253 − 18,012)	73.33 (65.12–82.30)	5,756 (4,747-6,995)	18.72 (15.43–22.74)	-4.09 (-4.41 to -3.78)
Central Europe	4,418 (3,638-5,460)	11.20 (9.22–13.84)	1,277 (872-1,850)	3.91 (2.66–5.66)	-2.81 (-3.24 to -2.37)
Central Latin America	76,045 (68,349 − 83,482)	139.76 (125.62-153.43)	35,211 (29,062 − 42,472)	40.89 (33.75–49.32)	-3.61 (-3.82 to -3.39)
Central Sub-Saharan Africa	153,719 (108,575 − 202,463)	930.64 (657.33-1225.74)	169,383 (120,040–232,144)	389.90 (276.32-534.37)	-2.06 (-2.37 to -1.76)
East Asia	125,963 (90,448 − 167,330)	30.85 (22.16–40.99)	18,997 (13,979 − 25,375)	4.38 (3.22–5.85)	-5.36 (-5.61 to -5.11)
Eastern Europe	16,522 (13,599 − 20,732)	22.98 (18.91–28.83)	5,234 (3,197-8,326)	8.55 (5.22–13.60)	-2.62 (-2.97 to -2.26)
Eastern Sub-Saharan Africa	470,623 (406,953 − 536,555)	813.24 (703.22-927.17)	411,016 (334,512 − 500,683)	293.55 (238.91-357.59)	-3.17 (-3.31 to -3.02)
High-income Asia Pacific	4,774 (3,779-6,014)	8.33 (6.59–10.49)	1,393 (951-2,012)	2.84 (1.94–4.10)	-3.66 (-4.1 to -3.23)
High-income North America	11,437 (8,401 − 15,916)	12.65 (9.29–17.60)	11,120 (7,956 − 14,920)	10.37 (7.42–13.91)	-0.32 (-0.51 to -0.13)
North Africa and Middle East	272,820 (219,474 − 324,251)	263.81 (212.23-313.54)	112,009 (84,543 − 145,990)	55.54 (41.92–72.39)	-4.78 (-4.97 to -4.59)
Oceania	2,858 (1,662-4,244)	141.32 (82.17-209.85)	5,144 (3,595-7,086)	116.13 (81.17-159.99)	-0.55 (-0.73 to -0.36)
South Asia	1,315,573 (1,116,161-1,497,912)	395.48 (335.53-450.29)	746,671 (559,140–976,528)	120.16 (89.98-157.15)	-4.00 (-4.19 to -3.81)
Southeast Asia	404,655 (338,524 − 473,087)	260.80 (218.18-304.91)	194,162 (158,026–242,903)	83.75 (68.16-104.77)	-3.37 (-3.44 to -3.29)
Southern Latin America	8,168 (6,944-9,670)	51.38 (43.68–60.83)	3,729 (2,812-4,885)	17.05 (12.86–22.33)	-2.79 (-3.02 to -2.56)
Southern Sub-Saharan Africa	41,239 (32,037–53,875)	238.18 (185.03-311.16)	31,625 (24,643 − 39,964)	115.45 (89.96–145.90)	-1.05 (-2.04 to -0.05)
Tropical Latin America	77,629 (69,713 − 86,526)	150.87 (135.49-168.16)	24,210 (20,615 − 27,912)	32.09 (27.32–36.99)	-4.06 (-4.48 to -3.64)
Western Europe	9,219 (7,253 − 12,289)	7.75 (6.10-10.33)	5,300 (3,362-8,270)	4.39 (2.79–6.85)	-1.36 (-1.51 to -1.2)
Western Sub-Saharan Africa	403,528 (314,574 − 491,338)	695.37 (542.08-846.69)	635,307 (496,632–820,756)	399.91 (312.62-516.64)	-1.57 (-1.65 to -1.49)

Abbreviations: ASDAR, age-standardized DALYs rate; DALYs, Disability-Adjusted Life Years; MHD, Maternal Hypertensive Disorders; SDI, sociodemographic index; GBD, Global Burden of Diseases, Injuries, and Risk Factors Study; EAPC, estimated annual percentage change; UIs, uncertainty intervals; CI, confidence interval

### MHD burden by age group: Temporal and regional trends

**Fig. 2:**
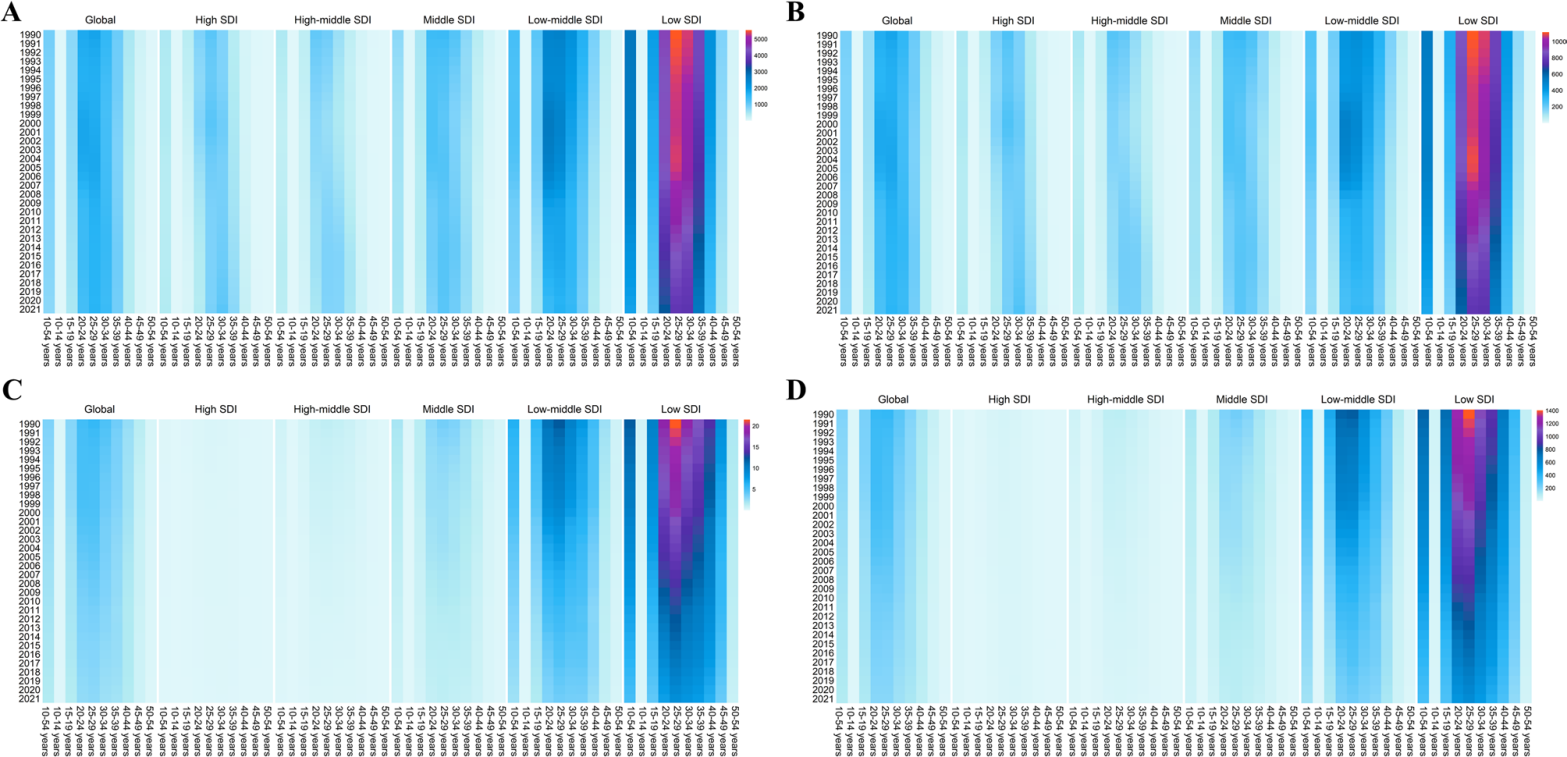
Heat map of burden trends in different age groups and regions. **A** The incidence rate of nine age groups (with 5-year intervals within 10–54 years) from 1990 to 2021, both globally and in five territories (ranging from low to high SDI); **B** The prevalence rate of nine age groups (with 5-year intervals within 10–54 years) from 1990 to 2021, both globally and in five territories (ranging from low to high SDI); **C** The deaths rate of nine age groups (with 5-year intervals within 10–54 years) from 1990 to 2021, both globally and in five territories (ranging from low to high SDI); **D** The DALYs rate of nine age groups (with 5-year intervals within 10–54 years) from 1990 to 2021, both globally and in five territories (ranging from low to high SDI). Abbreviations: DALYs, disability-adjusted life-years; SDI, sociodemographic index

From a temporal perspective, the global burden of Maternal Hypertensive Disorders (MHD) gradually declined between 1990 and 2021 (Fig. 2). However, in examining different SDI categories, heat maps reveal that among individuals aged 10–54, Low SDI regions bear the heaviest burden (Fig. 2), exhibiting the highest incidence, prevalence, mortality, and DALY rates (Supplementary Tables 8–11). From an age-structure standpoint, the global burden of MHD is primarily concentrated in the 20–24 and 25–29 age groups. In High and High-Middle SDI regions, MHD incidence and prevalence peak in the 25–29 and 30–34 age groups. In contrast, in Middle and Low-Middle SDI regions, incidence, prevalence, mortality, and DALY rates are highest among those aged 20–24 and 25–29. Notably, in Low SDI regions—especially for individuals aged 25–29—incidence, prevalence, mortality, and DALY rates are the highest (Fig. 2). Detailed country-specific results on MHD burden by age group, time period, and region can be found in Supplementary Tables 12–15.

### Cross-country inequality analysis

**Fig. 3:**
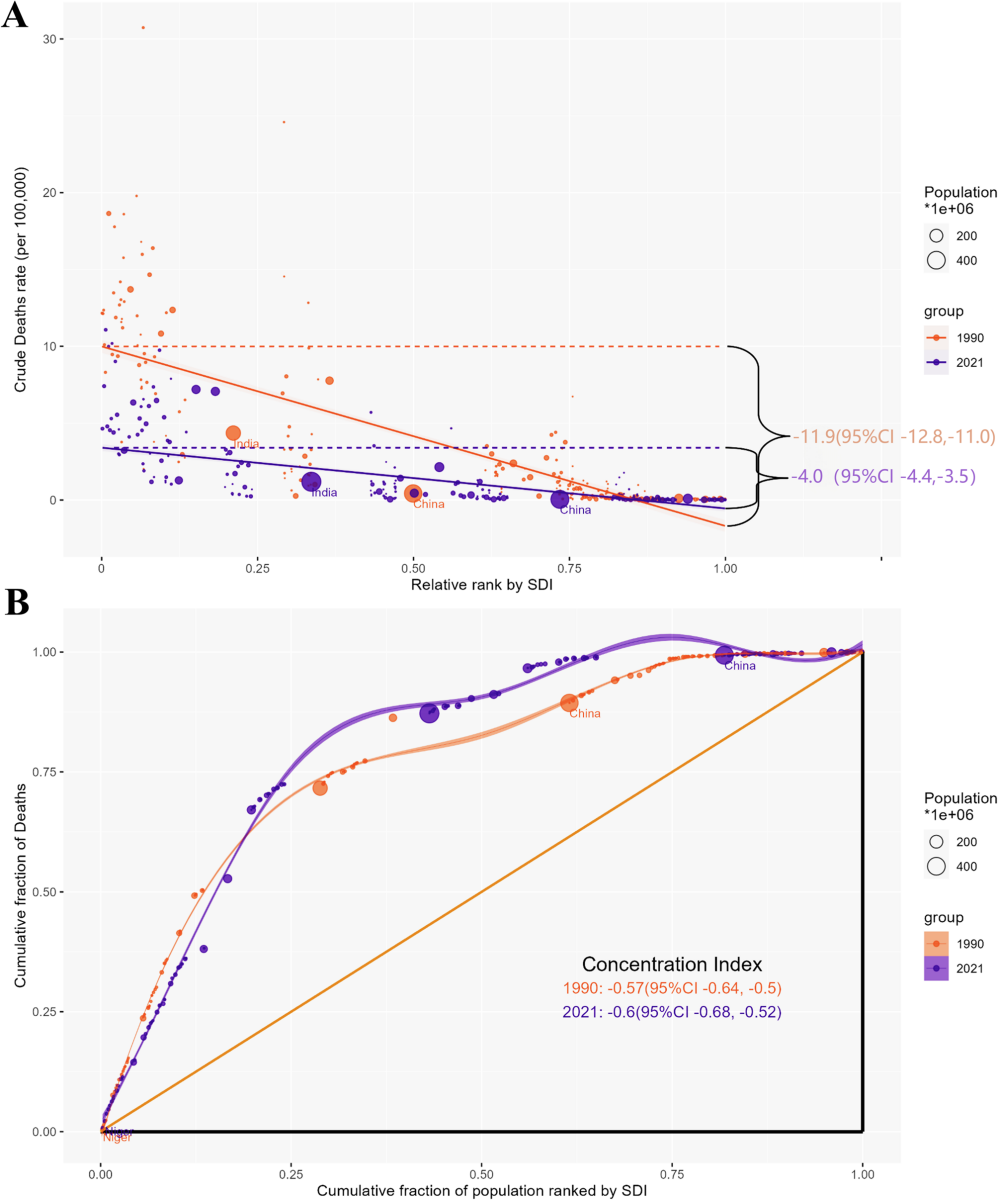
SDI-related health inequality regression (**A**) and concentration (**B**) curves for the Deaths of MHD worldwide, 1990 and 2021. Abbreviations: SDI, socio demographic index; MHD, Maternal Hypertensive Disorders

Significant absolute and relative inequalities associated with SDI were observed in the burden of Maternal Hypertensive Disorders (MHD), and these inequalities decreased markedly over time (Fig. 3). Interestingly, the number of deaths was disproportionately concentrated in countries with lower levels of social and demographic development. According to the slope index of inequality, in 1990, the difference in deaths per 100,000 population between countries with the highest and lowest SDI was − 11.90 (95% CI: -12.80, -11.00); by 2021, it had decreased to -4.0 (95% CI: -4.40, -3.50) (Supplementary Table 16). Furthermore, the concentration index—measuring relative gradient inequality—was − 0.57 (95% CI: -0.64, -0.50) in 1990 and − 0.60 (95% CI: -0.68, -0.52) in 2021, indicating an unequal distribution of MHD burden among countries with varying SDI levels.

### Risk factor analysis

**Fig. 4:**
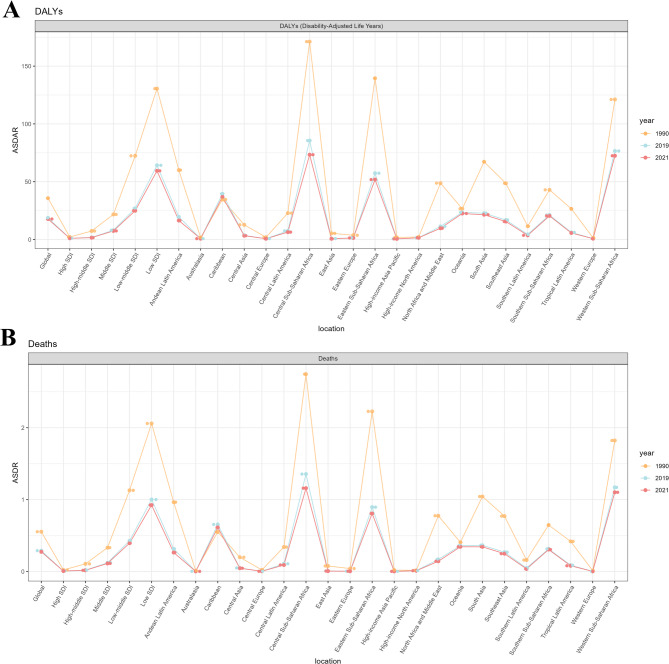
Trends in the rate of risk factors attributable to MHD in 27 regions around the world from 1990 to 2019 to 2021. **A** Trends in the rate of risk factors to age-standardized DALYs of MHD in 27 regions around the world from 1990 to 2019 to 2021. **B** Trends in the rate of risk factors to age-standardized death of MHD in 27 regions around the world from 1990 to 2019 to 2021. Abbreviations: DALYs, disability-adjusted life-years; MHD, Maternal Hypertensive Disorders

Among all potential risk factors identified in GBD 2021, global age-standardized Maternal Hypertensive Disorders (MHD) mortality and DALYs were primarily attributed to iron deficiency. The age-standardized mortality rate was 0.27% (95% CI: 0.13–0.39), and the age-standardized DALY rate was 0.46% (95% CI: 0.22–0.66) (Supplementary Table 17). Between 1990 and 2021, both the age-standardized mortality and DALY rates associated with iron deficiency showed a downward trend (Fig. 4A–B). The burden of iron deficiency was particularly severe in Low SDI regions, as well as in Central, Eastern, and Western Sub-Saharan Africa.

### Prediction model analysis

**Fig. 5:**
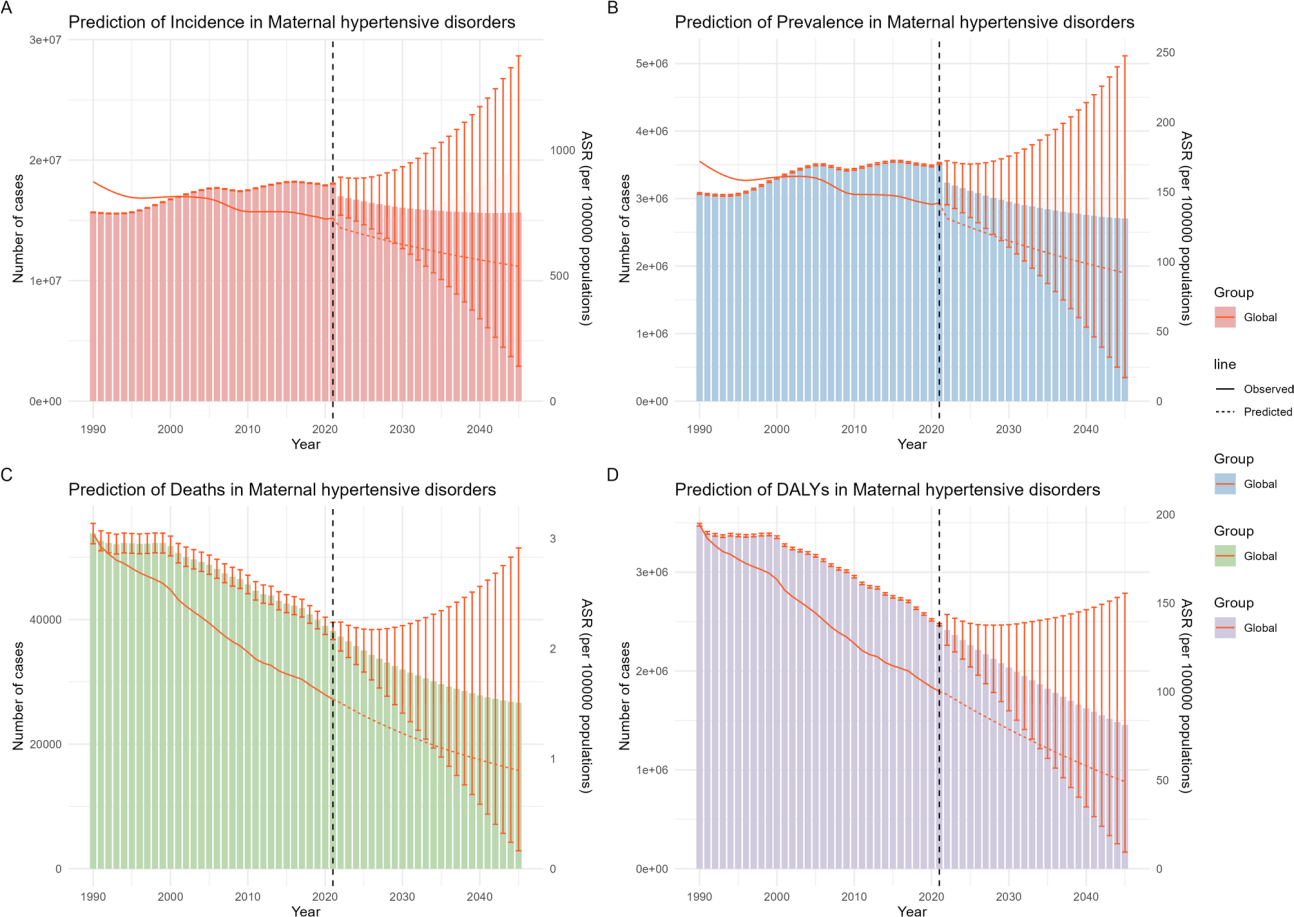
Projected the ASIR、ASPR、ASDR、ASDAR and the burden casess of MHD in global from 2022 to 2045 **A** The number of incidence casess and ASIR of MHD in global from 2022 to 2045. **B** The number of prevalence casess and ASPR of MHD in global from 2022 to 2045. **C** The number of deaths cases and ASDR of MHD in global from 2022 to 2045. **D** The number of DALYs cases and ASDAR of MHD in global from 2022 to 2045. Abbreviations: ASIR, age-standardized incidence rate; ASPR, age-standardized rate; ASDR, age-standardized deaths rate; ASDAR, age-standardized DALYs rate; DALYs, disability-adjusted life-years; MHD, Maternal Hypertensive Disorders

Figure 5 presents the predicted number of Maternal Hypertensive Disorders (MHD) cases in 2045, along with the age-standardized rates (ASR) for incidence, prevalence, mortality, and DALYs. Globally, the number of incidence, prevalence, mortality, and DALY cases is expected to decrease by 2045, and each corresponding ASR is projected to decline annually. Detailed global values for the number of cases and ASRs are provided in Supplementary Table 18. Supplementary Fig. 2 illustrates the projected number of cases by age group in 2045. The number of incidence and prevalence cases is forecast to decrease, primarily concentrated among individuals aged 20–24, 25–29, and 30–34. Likewise, mortality and DALYs are also expected to show a marked decline, concentrated mainly in the 20–24 and 25–29 age groups. For specific values, please refer to Supplementary Table 19.

### Two-Sample MR analysis

**Fig. 6:**
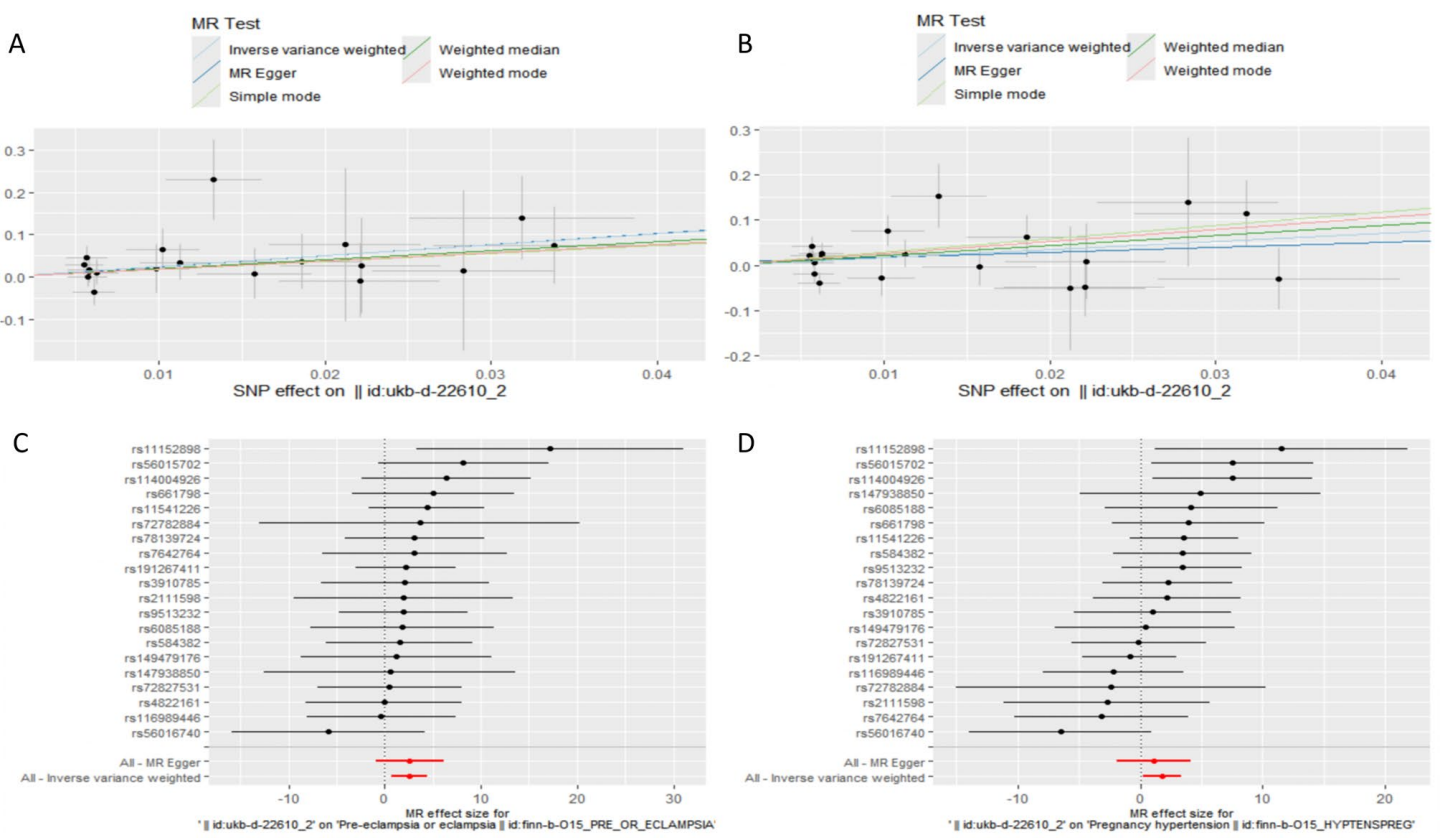
Scatter plot and forest plots showed the genetic association between occupational exposure factors and MHD. **A**, ** C** Workplace full of chemical or other fumes: Often on Pre-eclampsia or eclampsia. **B**, ** D** Workplace full of chemical or other fumes: Often on Pregnancy hypertension. Abbreviations: MHD, Maternal Hypertensive Disorders

We included nine occupational exposure factors to explore their causal relationships with six MHD outcomes. Notably, gestational hypertension served as the outcome for six different cohorts. After selecting the instrumental variables (IVs), we identified 2,317 independent SNPs associated with these occupational exposures (Supplementary Table 3). We then performed a comprehensive two-sample MR analysis to investigate the causal relationship between occupational pollution and maternal hypertensive disorders. Specifically, we used a range of MR methods—IVW, MR-Egger, weighted median, simple mode, and weighted mode—to evaluate the effects of particulate matter air pollution (PM2.5, PM10), gas or solid-fuel cooking/heating (an open solid fuel fire used regularly in winter), maternal smoking around birth, nitrogen oxides air pollution, working in an environment often filled with chemical or other fumes, heating type(s) in the home (oil/kerosene central heating), occasional pesticide exposure at work, and frequent exposure to very noisy workplaces. Our findings indicated a significant causal relationship between Workplace full of chemical or other fumes: Often and both preeclampsia/eclampsia as well as gestational hypertension (Table 5). The forest and scatter plots visually depict the notable associations between Workplace full of chemical or other fumes: Often and preeclampsia/eclampsia as well as gestational hypertension (Fig. 6). Using the IVW method, we observed an odds ratio (OR) of 13.08 (95% CI, 2.04–83.65) for preeclampsia/eclampsia, and an OR of 5.88 (95% CI, 1.23–28.06) for gestational hypertension. Conversely, our results showed no substantial causal relationship between gestational hypertensive disorders and PM2.5, PM10, gas or solid-fuel cooking/heating, maternal smoking around birth, nitrogen oxides air pollution, oil (kerosene) central heating in the home, occasional pesticide exposure at work, or frequent exposure to very noisy workplaces. Although IVW is an efficient estimator of causal effects when its assumptions hold, the results may be unreliable in the presence of significant horizontal pleiotropy, heterogeneity in the IV effect, weak IVs or outliers, or complex nonlinear relationships between exposure and outcome. To address these concerns, we conducted MR-Egger intercept tests for pleiotropy, and the results for both outcomes (preeclampsia/eclampsia and gestational hypertension) showed Egger P-values > 0.05, indicating no significant horizontal pleiotropy (Supplementary Table 20). Heterogeneity tests using both MR-Egger and IVW (Q statistics) also yielded P-values > 0.05, suggesting no notable heterogeneity(Supplementary Table 21). The overall symmetry of funnel plots confirmed this finding, suggesting minimal heterogeneity among the included SNPs (Supplementary Fig. 3A–B). Additionally, a leave-one-out analysis underscored the stability of the MR effect estimates (Supplementary Fig. 3C–D). Taken together, these results suggest that the estimated causal effects from the selected instrumental variables are credible, thereby reinforcing the robustness and interpretability of our findings.

**Table 5:** Mendelian randomization analysis identified several significant causal occupational exposure factors and maternal hypertensive disorders

Outcomes	Exposure	Method	Nsnp	SE	Pval	OR	95%CI
Pre-eclampsia or eclampsia	Workplace full of chemical or other fumes: Often	IVW	20	0.95	0.007	13.08	(2.04–83.65)
		IVW	20	1.80	0.165	13.5	(0.40-457.9)
		MR Egger	20	1.30	0.105	8.21	(0.64-104.42)
		Weighted median	20	2.01	0.368	6.39	(0.12-330.39)
		Simple mode	20	1.70	0.273	6.77	(0.24-188.26)
Pregnancy hypertension	Workplace full of chemical or other fumes: Often	IVW	20	0.80	0.026	5.88	(1.23–28.06)
		MR Egger	20	1.54	0.489	2.98	(0.14–61.30)
		Weighted median	20	1.03	0.032	9.03	(1.21–67.32)
		Simple mode	20	1.93	0.144	18.96	(0.43-831.88)
		Weighted mode	20	1.70	0.135	14.25	(0.51-401.56)

## Discussion

Based on the 2021 Global Burden of Disease (GBD) data, this study systematically analyzed the global epidemiological trends of maternal hypertensive disorders (MHD) from 1990 to 2021 and projected their future burden. To our knowledge, this is the first comprehensive epidemiological study that considers global burden trends, cross-national inequalities, risk factor assessments, predictive analysis, and, importantly, the causal relationship between occupational exposures and MHD. Our findings indicate that, worldwide, the incidence, prevalence, mortality, and disability-adjusted life years (DALYs) of MHD have generally declined. However, in certain countries and regions, these indicators deviate from the global trend, underscoring the need for targeted public health strategies in these priority areas.

From the perspective of the Social Demographic Index (SDI) distribution, the MHD burden is mainly concentrated in regions with lower SDI (Low SDI). High SDI regions show peak incidence and burden at 30–34 years old, whereas Low SDI regions reach similar levels earlier, at 25–29 years. Cross-national inequality analyses reveal that, between 1990 and 2021, the slope index of inequality decreased significantly, suggesting that the uneven burden of MHD across different countries has improved. Iron deficiency remains the leading risk factor for MHD, posing the greatest challenge in Low SDI areas. Bayesian age-period-cohort (BAPC) model projections indicate that, by 2045, incidence, prevalence, mortality, and DALY cases of MHD will continue to decline, with decreasing age-standardized rates (ASRs). Furthermore, two-sample Mendelian randomization analysis shows a significant causal association between frequent exposure to chemicals or fumes in the workplace Workplace full of chemical or other fumes: Often and MHD, offering new evidence and perspectives on the role of occupational exposures as a primary risk factor for MHD.

Similar to previous GBD studies, analyses from 1990 to 2019 also suggested marked decreases in the age-standardized death rate (ASDR) and age-standardized DALY rate for MHD [26]. The cross-national inequality analysis demonstrates that the slope index of inequality has significantly declined from 1990 to 2021, reflecting an overall improvement in the uneven distribution of MHD burden among countries. This achievement can be attributed to the development and prioritization of the World Health Organization’s maternal health programs over the past three decades, as well as the continual progress of large-scale clinical research supported by various governments [27– 31]. These efforts have contributed to more effective primary prevention and improvement in mortality and DALY rates. Nevertheless, some regions—namely Central Asia, Eastern Europe, and Western Europe—have seen rising incidence and prevalence of MHD, while the Caribbean region has experienced increasing mortality and DALYs. Overall, the surge in incidence and prevalence of MHD in Central Asia, Eastern Europe, and Western Europe, as well as the rise in mortality and DALYs in the Caribbean, can be traced to multiple interacting factors. These include an aging population and increased pregnancies at advanced maternal age, lifestyle changes and rising obesity, a growing burden of chronic diseases, improved diagnostic and monitoring capacities, disparities in healthcare resources and medical conditions, and shifts in regional demographics [32, 33]. In response to the increase in prevalence in some regions (Central Asia, Eastern Europe, and Western Europe), we emphasize the need for enhanced early screening and prenatal risk stratification, which may help identify and manage maternal hypertensive disorders (MHD) before complications arise. The increase in prevalence may also reflect improved detection rather than an increase in burden, which requires nuanced interpretation and response. For regions where mortality and disability-adjusted life-year (DALY) rates are increasing but remain high (the Caribbean), we recommend targeted interventions to improve access to emergency obstetric care, ensure the availability of antihypertensive medicines during pregnancy, and train health workers in the early recognition and management of pre-eclampsia and eclampsia. Previous studies indicate that, in Latin America and the Caribbean, hypertensive disorders account for about 26% of maternal deaths, with 10% of these deaths linked to eclampsia [34]. This figure is noticeably higher than the 9% reported in Africa and Asia. Although maternal mortality in high-income countries is substantially lower than in developing regions, 16% of maternal deaths are still attributable to hypertensive disorders [35]. Moreover, high SDI regions generally exhibit higher economic and cultural levels, leading to more frequent pregnancies at advanced maternal age [36], whereas this trend is less pronounced in low SDI regions. These observations help explain why, in our results, incidence and prevalence rates in high SDI areas shift from the 25–29 age group to 30–34, whereas they remain predominantly within the 25–29 range in low SDI regions.

Quantifying cross-national inequalities in the burden of MHD across different SDI gradients could illuminate distribution patterns and highlight areas for improvement in prevention and control. It is widely accepted that high-SDI countries have better access to healthcare systems, which makes them more efficient—factors that may reduce the burden of disease. The concentration of MHD burden in low-SDI countries stems from two key issues. First, the risks associated with iron deficiency are most severe in these regions; second, low-SDI countries face enormous challenges due to population growth and insufficient health resources. To this end, we recommend expanding access to iron supplements and dietary fortification programmes for women of childbearing age. Integrate nutritional assessment and counselling into routine antenatal care in response to the World Health Organization’s global initiative to halve the prevalence of anaemia by 2025. Establish national health surveillance systems that can break down data by SDI, geography and age group to monitor trends are recommended. International support and collaboration are encouraged, particularly through WHO and UN initiatives to allocate resources and technical assistance to high-burden, low-SDI areas. In addition, we note that while the incidence and prevalence rates in other SDI categories (e.g., low-SDI and high-SDI regions) have declined significantly, the lack of significant changes in the incidence and prevalence rates in high-middle SDI regions and Andean Latin America may reflect multiple factors, such as: Stable access to and coverage of health care: Health system improvements in these regions may have reached a plateau, resulting in neither a significant increase nor a significant decrease in the detection or reporting of incidence. For example, in the Andean Latin America region, while national policies (maternal health programs) may have stabilized results, persistent local disparities—particularly in rural or indigenous communities—may mask local increases in morbidity or mortality. Sociodemographic transitions: Changes in maternal age, urbanization, and occupational patterns may offset each other in terms of risk factor burden. For example, in high-middle SDI regions, moderate improvements in health care (access to prenatal care) may be offset by rising risk factors such as delayed childbearing, obesity, or occupational exposures. Data quality limitations: Fluctuations in surveillance or registry completeness may dampen trends in some subregions. Accordingly, we recommend: Continue to invest in maternal health surveillance systems to improve trend sensitivity. In the Andean Latin America region, expand community prenatal services in underserved areas to address geographic inequalities, and strengthen nutrition programs, prioritizing iron supplementation and dietary support, given that iron deficiency is a major risk factor for MHD worldwide. Policy priorities should be to develop prevention strategies targeting occupational and environmental risk profiles, especially in urbanized high-middle SDI regions. Targeted occupational hazards can be reduced, and workplace safety regulations can be enforced to reduce exposure to chemical fumes, which are modifiable risk factors highlighted by our MR results. and incorporating obesity and hypertension screening into routine prenatal care to mitigate compounding metabolic risks.

Conduct targeted research on region-specific risk factors that may not be captured by current GBD models. These strategies align with our research’s emphasis on tailored interventions—combining risk-targeted mitigation measures and strengthening systemic health care—to address stagnation in regions where trends are not evident.

Within the GBD 2021 database, iron deficiency stands out as the primary risk factor for MHD. As in the GBD 2019 findings, reductions in iron deficiency–attributable ASDR, age-standardized DALYs, and years of life lost (YLL) have been notable among maternal diseases, including hypertensive disorders in pregnancy [26]. Research by Woodman et al. showed that perinatal iron deficiency combined with a high-salt diet can result in sex-dependent, long-term mitochondrial dysfunction and oxidative stress in the kidneys, potentially elevating the risk of hypertension and cardiovascular diseases [37]. However, large-scale clinical studies remain necessary to determine effect size and intervention efficacy in the broader population. In 2023, the WHO called for accelerated action to reduce iron deficiency–related anemia in pregnant women and introduced its first comprehensive framework for reducing anemia, launched at the International Maternal and Child Health Conference. The WHO urges countries to act quickly, aiming to halve the prevalence of anemia among women of reproductive age by 2025. In line with this target, public health policies should incorporate screening and management of iron deficiency and high-salt diets into routine prenatal checkups and nutritional counseling. As GBD data updates continue and basic research further illuminates the mechanisms linking perinatal nutrition and disease, clinical and public health efforts must keep emphasizing iron deficiency prevention and dietary interventions to lower maternal morbidity and diminish chronic disease risk for future generations.

This study employed a Bayesian age-period-cohort (BAPC) model based on GBD data from 1990 to 2021 to project the global burden of MHD from 2022 to 2045. The results suggest that, over the next three decades, the age-standardized rates (ASR) of incidence, prevalence, mortality, and DALYs for MHD will likely decline annually, alongside overall reductions in these four indicators. With continued advancements in diagnostic capabilities and relevant interventions, the MHD burden is expected to decrease further. Several points, however, require consideration: Suitability of BAPC: BAPC incorporates age, period, and cohort effects, allowing a more nuanced analysis of disease dynamics over time. Compared to traditional age-period-cohort analyses, the Bayesian approach partially mitigates the identifiability problem and manages uncertainties more effectively over a long projection horizon. Nonetheless, BAPC remains sensitive to model assumptions and priors; inappropriate choices may introduce bias [38]. Exogenous Shocks: These projections rely on WHO population estimates to 2050. Major policy changes, improvements in healthcare services, or major global events (e.g., pandemics, wars, economic or occupational crises) could alter future trends. Population Demographics: While population aging drives higher incidence and mortality for some chronic diseases, shifts in fertility rates and disease profiles might reduce the overall share of MHD in future populations [39]. Dynamic Monitoring and Model Adjustment: In a rapidly changing global health landscape marked by emerging infectious diseases, occupational changes, and public health emergencies, regular data updates and model recalibrations are crucial for accurate projections. Overall, using the BAPC approach with GBD data, our study is the first to forecast a year-by-year global burden for MHD from 2022 to 2045. The “optimistic” trend of declining rates suggests that, if the current effective healthcare interventions and public health measures are sustained and expanded, the global burden of MHD could be further reduced. Nonetheless, caution is advised in interpreting and applying these projections, as they remain subject to model assumptions, data limitations, and unforeseen external factors.

In addition, this study used Mendelian randomization (MR) to explore potential causal relationships between occupational pollutants and MHD through genome-wide association study (GWAS) data, thereby complementing existing research on occupational exposures as a risk factor for hypertensive disorders in pregnancy. Our findings suggest no causal relationship between PM2.5 exposure and MHD, whereas “Workplace full of chemical or other fumes: Often” is significantly associated with MHD. This contradicts some earlier research, which consistently identified significant correlations between PM2.5 and MHD [40–44]. Traditional epidemiological studies typically rely on occupational or individual monitoring data, which may introduce measurement error and bias. Variations in exposure windows (e.g., early vs. mid pregnancy) may also affect findings. Compared to PM2.5, exposure to chemicals or fumes in the workplace is often more concentrated, more intense, and more consistently accumulated during working hours. Moreover, GWAS data for such workplace exposures often include clearer questionnaire- or phenotype-based information (e.g., job type, frequency of exposure). Consequently, in the setting of “intense and specific occupational exposure,” genetic instruments may more robustly capture the relationship between exposure and outcomes, making it easier to detect causal associations via MR. However, our results also show that the 95% confidence interval for the association between “Workplace full of chemical or other fumes: Often” and MHD is extremely wide, indicating a high degree of uncertainty. Although the finding is statistically significant, its clinical interpretation warrants caution. A lower bound of 2.04 suggests at least a weak positive correlation, whereas an upper bound of 83.65 reflects a high degree of imprecision—one of the main limitations of this study. Future research could consider additional statistical methods or stratified analyses to minimize confounding influences. Our results suggest a potential causal relationship but do not provide definitive evidence and should be interpreted in the context of genetic and environmental parameters of the study population.

## Limitations

Our research faces several limitations. Data sparsity remains a challenge in many low-income or conflict-affected settings, where estimates rely more on models than on empirical evidence. Misclassification bias or differences in diagnostic criteria (e.g., preeclampsia vs. gestational hypertension) can affect comparability across settings. The modeling framework assumes stationary covariate effects over time, which may not fully capture changing health care access, diagnostic practices, or sociodemographic changes. Besides, the use of age-standardized rates may mask important subnational or age-specific patterns that are policy-relevant but obscured in aggregate estimates. GBD estimates rely heavily on aggregated data from hospital records, health surveys, and demographic data, which may vary in completeness, diagnostic accuracy, and temporal consistency across regions. This may lead to systematic underreporting, especially in resource-poor settings, thereby potentially underestimating the burden in low-SDI countries and exaggerating inequality indicators. We hereby explicitly state that caution is warranted when interpreting regional comparisons based on model estimates. Because GBD data are aggregated at the aggregate level and parity is not tracked separately, it is indeed possible that some women contribute to the incidence count more than once in different pregnancies, which may slightly increase the burden. In our Mendelian randomization section, we noted that instrumental variables derived from genome-wide association studies (GWAS) of European ancestry may not be fully generalizable to global populations. In addition, pleiotropy or weak instrument bias may shift causal estimates in either direction. Although sensitivity analyses support the validity of our instruments, we now explicitly state that MR results should be interpreted with caution, especially in multiethnic or underrepresented populations, and large-scale cohort studies are necessary to validate these findings for clinical and public health applications.

## Conclusion

Drawing on the GBD 2021 database, this study systematically examined maternal hypertensive disorders (MHD) from multiple angles—global burden trends, cross-national inequalities, risk factor assessments, and future projections—covering the period from 1990 to 2021. Overall, MHD incidence, prevalence, mortality, and DALYs have declined worldwide, but certain regions (such as Central Asia, Eastern Europe, Western Europe, and the Caribbean) continue to show sustained increases. Our two-sample Mendelian randomization analysis further revealed a potential causal link between frequent chemical or fume exposures in the workplace and MHD, warranting further investigation into their role in maternal health outcomes. We urge global public health policymakers to reinforce iron deficiency prevention, promote nutritional and high-salt diet interventions during pregnancy, and systematically monitor workplace exposures. Strengthening these primary prevention and precision interventions can help build on existing achievements and reduce the global burden of hypertensive disorders in pregnancy.

## Electronic supplementary material

Below is the link to the electronic supplementary material.

Supplementary Material 1

Supplementary Material 2

Supplementary Material 3

Supplementary Material 4

## Data Availability

All data used in this study can be performed using published GWAS summary statistics and GBD 2021 portal (http://ghdx.healthdata.org/gbd-2021). In the prediction analysis, population estimates were obtained from the 2019 revision of the United Nations World Population Prospects, disaggregated by year (up to 2100), age, and sex (https://population.un.org/wpp/downloads). Exposure data on occupational pollutants and MHD case data were obtained from the UK Biobank and FinnGen databases, respectively, GWAS summary statistics on MHD were derived from the FinnGen consortium and downloaded from (https://www.finngen.fi/en). GWAS summary statistics of instrumental variables were obtained from (https://gwas.mrcieu.ac.uk/), and source data of the GWAS are available in the supplementary tables of the article.
