# Supplementary figures and images for: Global burden of maternal hypertensive disorders (1990–2045): trends, regional disparities, and causal links to occupational exposures
Source: BMC Pregnancy Childbirth. 2025 Jun 2;25:641. doi: 10.1186/s12884-025-07766-y (PMC12128521; doi:10.1186/s12884-025-07766-y)

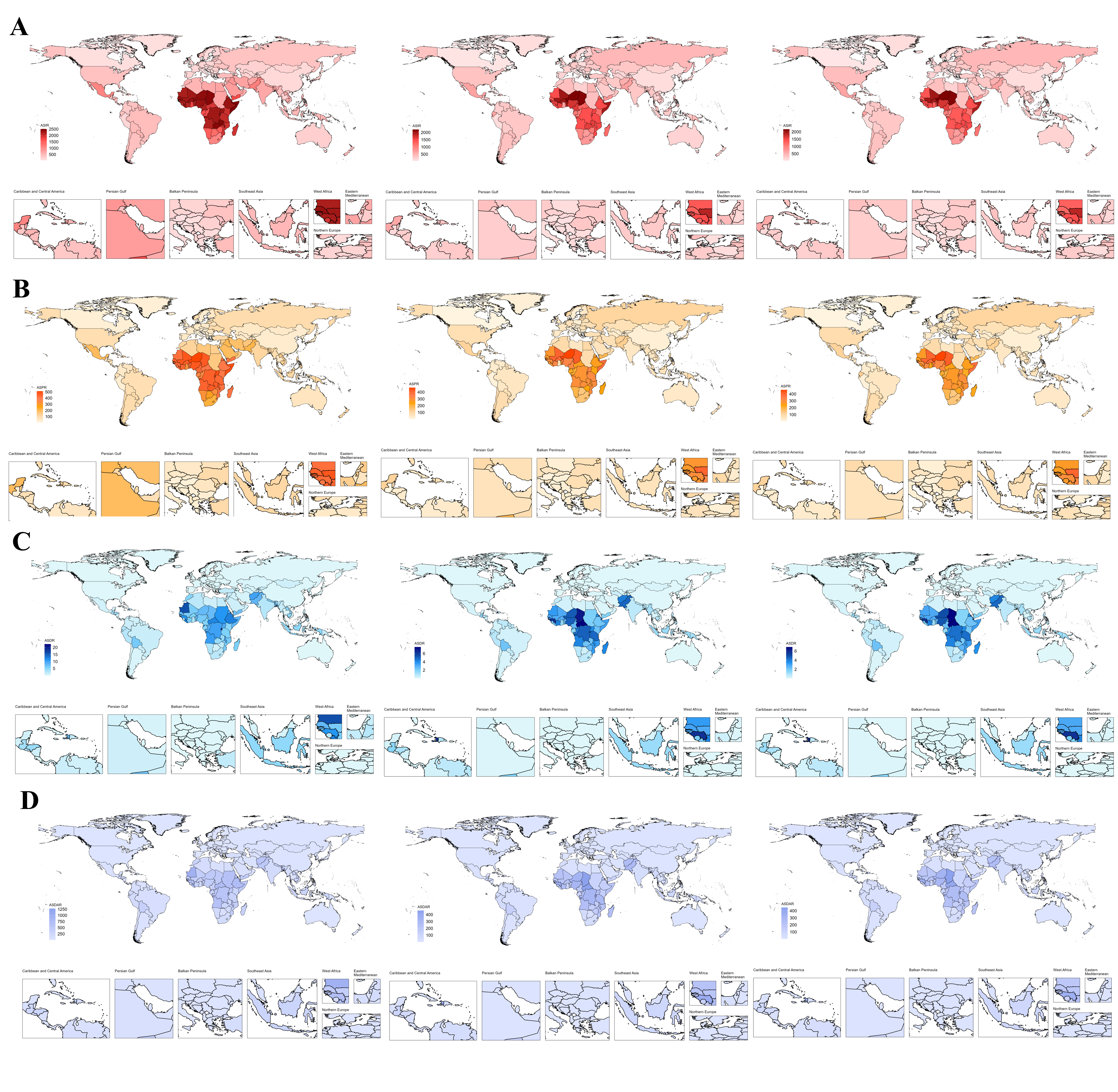

Supplement: Supplementary file 1 — Supplementary Material 1 [file 12884_2025_7766_MOESM1_ESM.tif]

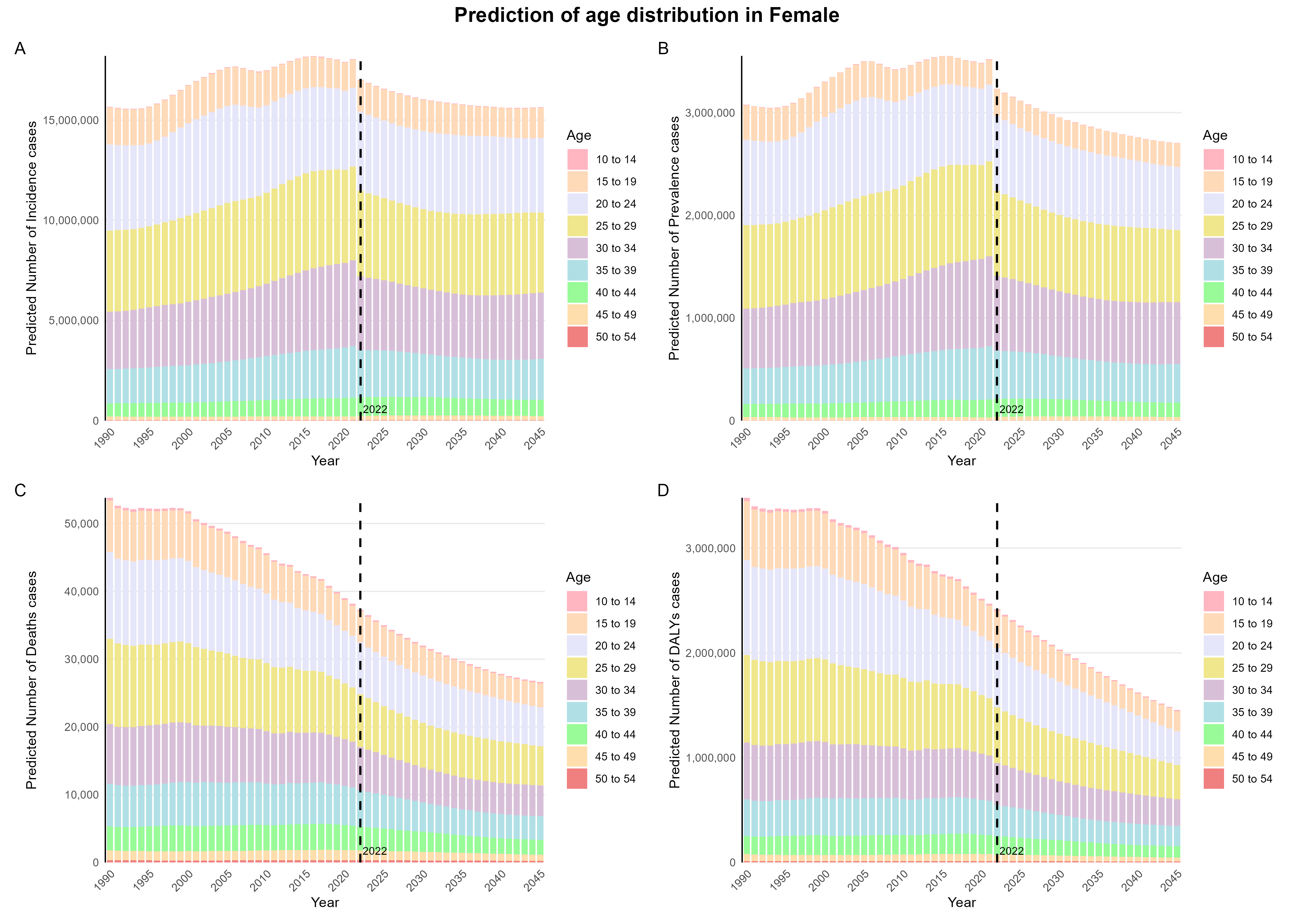

Supplement: Supplementary file 2 — Supplementary Material 2 [file 12884_2025_7766_MOESM2_ESM.tif]
